# solQTL: a tool for QTL analysis, visualization and linking to genomes at SGN database

**DOI:** 10.1186/1471-2105-11-525

**Published:** 2010-10-21

**Authors:** Isaak Y Tecle, Naama Menda, Robert M Buels, Esther van der Knaap, Lukas A Mueller

**Affiliations:** 1 Boyce Thompson Institute for Plant Research, Tower Rd, Ithaca, NY 14853, USA; 2 Department of Horticulture and Crop Science, The Ohio State University/OARDC, Wooster, OH 44691, USA

## Abstract

**Background:**

A common approach to understanding the genetic basis of complex traits is through identification of associated quantitative trait loci (QTL). Fine mapping QTLs requires several generations of backcrosses and analysis of large populations, which is time-consuming and costly effort. Furthermore, as entire genomes are being sequenced and an increasing amount of genetic and expression data are being generated, a challenge remains: linking phenotypic variation to the underlying genomic variation. To identify candidate genes and understand the molecular basis underlying the phenotypic variation of traits, bioinformatic approaches are needed to exploit information such as genetic map, expression and whole genome sequence data of organisms in biological databases.

**Description:**

The Sol Genomics Network (SGN, http://solgenomics.net) is a primary repository for phenotypic, genetic, genomic, expression and metabolic data for the Solanaceae family and other related Asterids species and houses a variety of bioinformatics tools. SGN has implemented a new approach to QTL data organization, storage, analysis, and cross-links with other relevant data in internal and external databases. The new QTL module, solQTL, http://solgenomics.net/qtl/, employs a user-friendly web interface for uploading raw phenotype and genotype data to the database, R/QTL mapping software for on-the-fly QTL analysis and algorithms for online visualization and cross-referencing of QTLs to relevant datasets and tools such as the SGN Comparative Map Viewer and Genome Browser. Here, we describe the development of the solQTL module and demonstrate its application.

**Conclusions:**

solQTL allows Solanaceae researchers to upload raw genotype and phenotype data to SGN, perform QTL analysis and dynamically cross-link to relevant genetic, expression and genome annotations. Exploration and synthesis of the relevant data is expected to help facilitate identification of candidate genes underlying phenotypic variation and markers more closely linked to QTLs. solQTL is freely available on SGN and can be used in private or public mode.

## Background

Many economically important traits are quantitatively inherited, influenced by the environment and controlled by many genes of small and large effect [1-4]. A powerful approach to understanding the genetic basis of such complex traits is through identification of quantitative trait loci (QTL) associated with the trait [1,5,6]. Once QTLs are identified, generally, confirmation and fine mapping of the QTLs is required, followed by map-based cloning or a candidate gene approach that, in turn, necessitates further functional analysis of the candidate genes through expression profiling, genetic complementation and/or insertional mutagenesis [2,3,7,8]. Fine mapping of QTL requires mendelization of the locus [9] and large populations. This effectively means several generations of backcrosses to create nearly isogenic lines (NILs), which is a costly and time consuming effort [10,11]. Furthermore, dissecting the quantitative trait nucleotide (QTN) variation and understanding the molecular and physiological basis underlying the phenotypic variation remains a great challenge [3,12]. A step forward in identifying candidate genes and dissecting the QTN(s) would be to develop tools to harness the genetic resources as well as expression and whole genome sequence data of organisms in biological databases through bioinformatics approaches [3,11-13].

The Sol Genomics Network (SGN, http://solgenomics.net; [14]) is a repository for phenotypic, genetic, genomic, expression and metabolic data for the Solanaceae and other related Asterids families and houses a variety of bioinformatic tools. SGN has added a new QTL module, solQTL http://solgenomics.net/qtl/, to its repertoire of bioinformatics resources. The solQTL module presents a new approach to QTL data organization, storage, and analysis. Additionally, the module allows dynamic cross-linking with other relevant data including to the annotated genome sequence, expression data and genetic maps found in internal and external databases. The solQTL module employs a user-friendly web interface for uploading raw phenotype and genotype data, R/QTL mapping software (http://www.rqtl.org/[15]), an add-on package to the R statistical software, for on-the-fly QTL analysis and algorithms to cross-reference QTLs to relevant datasets.

In primary mapping populations, QTL regions may span up to 30 cM [12] containing thousands of genes [3,16]. However, some studies have revealed that QTLs and genes of quantitative traits can be identified from within 0 to 1.9 cM for major QTLs, which explain >25% of the phenotypic variation, (e.g. fw2.2, [17]; ovate, [18] in tomato) and 0 to 3 cM for small QTLs (<25%) (Hd1, Hd2, and Hd3 in rice [19]) from the peak marker [10]. Price (2006), in his review article, implied that searches for candidate genes of small QTLs can be done within 1 - 2 cM on either side of the mean QTL position in a primary QTL population, especially when fine mapping is problematic but datasets from multiple studies can be used. However, it is known that genes or QTNs that affect the QTL may be tens of kilobases (kbs) away from the mean QTL position or in genes with unrelated or regulatory functions [1,3,12,20]. Nonetheless, exploration and synthesis of genetic, genomic and expression data on corresponding regions around the peak QTL and within region delimited by flanking markers is useful, and can yield data leading to identification of candidate genes for the QTL of interest.

SGN, as a primary data and bioinformatics center for the Solanaceae, has the database structure, information and comparative analysis tools at hand to facilitate the cross-referencing of QTLs with other internal and external datasets. The SGN database houses the tomato genome sequence, *Solanum lycopersicum *cv Heinz 1706, including annotations provided by the International Tomato Genome Annotation Group (ITAG) [21]. SGN also hosts whole genome sequences of *S. tuberosum *cv DM1-3 516R44 (currently BAC-based sequencing only) and *S. pimpinellifolium *cv LA1589. With the on-going efforts to sequence 100 more Solanaceae species (SOL-100 project, [22]) and the SNP database under development at SGN (unpublished), we envision that anchoring QTLs to the genomes will open additional windows in facilitating the elucidation of the molecular basis of complex traits and may lead in identification of QTL markers for efficient marker-assisted breeding.

Currently, major plant model organism databases such as Gramene (http://www.gramene.org[23,24], MaizeGDB (http://www.maizegdb.org[25], and animal databases such as Animal QTLdb (http://www.animalgenome.org, [26,27]) rely on in-house curators or the research community to curate QTL mapping outputs from published literature. A problem with QTL data is that historically, a large part of the original data has been lost, as the raw data (phenotypic and genotypic scores of the populations) were not recorded in the publications. It is therefore impossible to evaluate many of these described QTLs independently and retroactively. The solQTL module is unique in that (1) it uses a user-friendly, guided, step-wise, web interface to allow researchers to upload their raw phenotype and genotype data; (2) researchers can choose the statistical parameters for the on-the-fly, real-time, QTL mapping; (3) users can opt to make their raw and analyzed data either private or public; and (4) as growing number of journals are requiring authors to deposit their data in public databases prior to publication of their work, solQTL could facilitate this for the Solanaceae research community, if it was made a requirement for QTL data. Importantly, this system stores all the primary data for the identification of a QTL, such that the data can be re-evaluated in the future.

Here, we describe the development of the solQTL tool and demonstrate its application.

## Construction and content

### Data submission

The researchers upload their raw phenotype and genotype data into the solQTL database and select the statistical parameters for the QTL mapping and visualization. A step-by-step, user-friendly, web interface (Additional file 1) allows users to upload their QTL population details such as name, description, and parents, trait data (description, definition and units of measurement), phenotypic and genotypic data separately in a tab-delimited files. To allow cross-referencing QTL markers to relevant datasets in SGN, markers used in the mapping population must exist in the SGN marker database. Users are prompted to set the statistical parameters they would like to apply for the QTL analysis. The statistical options available at this stage are described in the 'QTL Statistical Analysis' section below. Users have the option to save their data in the database, for either public or private use. The public option allows all users to see the dataset, whereas the private option only lets the data owner and SGN curators access the data. For private data, the population name, description, and contact information of the data owner are publicly accessible.

Built-in validation algorithms check for mandatory fields that need to be filled in and verify data formatting; for the statistical parameters, the consistency of the selection of the parameters is also validated. For example, the combination of QTL mapping method and QTL genotype probability estimation method selected is checked. Appropriate informative messages are displayed to the user when a required data field is empty or filled with incompatible information. To assist users in the use of the tool, a link to the guideline document is available at each step in the uploading and analysis process http://solgenomics.net/qtl/guide.pl.

### Database content

Currently, the solQTL database holds raw QTL data for three F2 intercross tomato QTL populations [28-30] submitted by Esther van der Knaap of Ohio State University. For each population, 18 - 46 fruit morphology related traits are evaluated for QTLs.

### QTL statistical analysis

The solQTL module uses R/QTL for the QTL mapping analysis. Among the QTL mapping functions presently implemented using the R/QTL in the solQTL tool include a one-dimension single-QTL scan model along with the single marker analysis and interval mapping methods. With the single-QTL model, the genome is scanned at a self-determined genome size, one at a time, for the presence of a QTL [31]. Genome scan sizes of 0 (marker regression), 1, 2.5, 5 or 10 cM can be selected. Users can choose between QTL mapping methods such as standard interval mapping (Maximum Likelihood with EM algorithm [32,33]), Haley-Knott regression [34] and Multiple Imputation [35]. For the Multiple Imputation QTL mapping method and the QTL genotype probability estimation using Simulation, one can select either 5, 10, 15, or 20 imputations to run. The underlying algorithm for calculating the QTL genotype probabilities and for dealing with missing or partially missing genotype data is hidden Markov model (HMM) technology [36]. A permutation test [37] for determining the LOD threshold significance level is also implemented. Users have the option to run 100 or 1000 permutation tests or none at all. QTL confidence interval (flanking markers) is determined based on a 95% Bayesian Credible Interval method with the interval expanded to the nearest markers [35,38]. Based on the highest LOD score in the linkage group, a peak marker for a QTL is identified using R/QTL's 'find.marker' method. When a marker is not located at the peak QTL, the nearest possible marker is identified [15].

Currently, solQTL uses genetic map data from *a priori *computation and thus users need to provide linkage map data with the genotype data file, as indicated in documentation provided with the tool http://solgenomics.net/qtl/guide.pl. The linkage map plotting and visualization, however, is done using Perl modules developed both in-house and from the open sources community via the Comprehensive Perl Archive Network (CPAN, http://cpan.org).

At this stage, the solQTL is available only for backcross and F2 intercross diploid populations for traits with continuous phenotypic variation. As the primary focus of solQTL is to link the predicted QTLs to annotated whole genome sequences, dense reference genetic maps and other relevant expression data at SGN using tools such as the SGN Comparative Map Viewer [39] and genome browser (GBrowse), the tool is designed for use at SGN and hence, applicable and most useful for organisms that are supported at SGN.

### Software development

SGN currently uses PostgreSQL http://www.postgresql.org version 8.3 for the back-end database. The website is implemented in Perl http://www.perl.org version 5.10.0. In-house and externally developed Perl modules are used for organizing, formatting and communicating data to R for the statistical analysis, the visualization of the QTL mapping output on the browser, and the cross-linking of QTLs to relevant datasets on SGN. A 1.08-56 version of the R/QTL (http://www.rqtl.org, [15]) is implemented on a 2.7.1 version of the R statistical software (http://www.r-project.org, [40,41]).

## Utility

### QTL mapping and visualization

Once a user successfully uploads the prerequisite data in the supported format and sets the statistical parameters, the respective QTL population detail page is displayed (Additional file 2). For the purpose of demonstrating the QTL analysis, output visualization and cross-referencing to relevant genetic and genomic datasets with solQTL, we use raw data from the F2 population of *Solanum lycopersicum *cv. Howard German x *S. pimpinellifolium *accession LA1589 http://solgenomics.net/phenome/population.pl?population_id=12, and specifically the trait "Fruit Area" [29]. The statistical parameters used for the particular QTL mapping output are in the corresponding legend. However, it should be noted that validation of the predicted QTL for the trait is beyond the scope of this work. On the population webpage, the name and description of the population is displayed along with a list of the traits phenotyped with the corresponding descriptive statistics. Also on the same page are any publications that document the QTL data, if available, and links to download the public raw phenotype and genotypic data of the QTL population.

**Figure 1:**
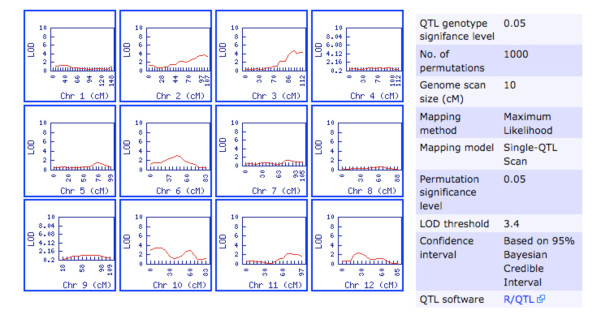
**Genome-wide QTL mapping output**. An example of genome-wide QTL mapping output from solQTL for a trait called 'Fruit Area' (see supplemental figure 2) and legend detailing the analysis parameters (Source: http://solgenomics.net/phenome/population_indls.pl?population_id=12&cvterm_id=39945/). Clicking a linkage group with a significant QTL (eg. Chr 3) leads to the QTL detail page (see Figure 2).

**Figure 2:**
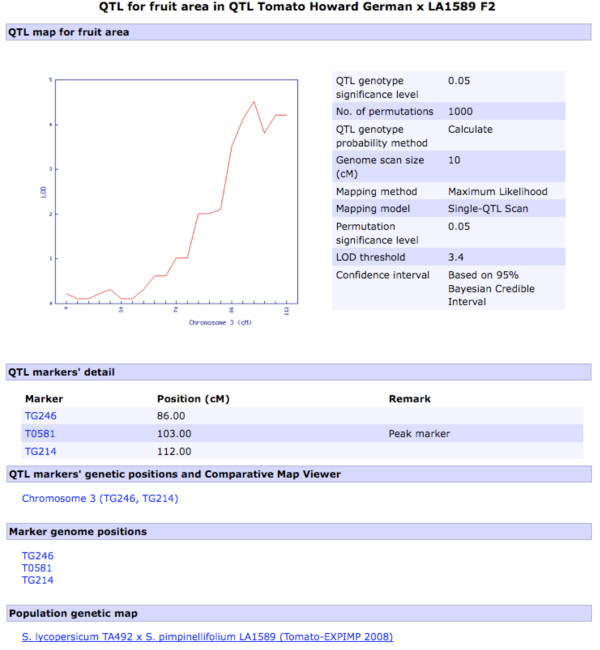
**A QTL detail page**. An example QTL detail page, e.g., for putative 'Fruit Area' QTL (see figure 1), showing the analysis details, links to detail pages of QTL flanking and peak markers, the QTL's linkage group page and Comparative Map Viewer page (see Figure 3), and genome positions of the markers (see Figure 4).

The QTL analysis is performed dynamically on a trait-by-trait basis when the user visits the trait of interest on the QTL population's webpage. On the QTL population's page, under the QTL(s) heading, clicking the graph icon corresponding to the trait of interest initiates the QTL mapping analysis. The analysis output is displayed on a new trait page where one can view its QTL analysis output (Figure 1) and frequency distribution of the phenotype data for the trait. QTL mapping locations from the output of the QTL analysis are viewable for the whole genome on a per-linkage group basis. The details of the analysis are displayed in a legend including genome-wide LOD threshold significance level, if the user opted for permutation test and probability level, size of genome scan and method used for the calculation of the QTL genotype probabilities, QTL mapping method and QTL model used. Clicking a chromosome with a QTL, after visually determining the chromosome with a significant QTL relative to the LOD threshold value in the legend, takes the user to a QTL detail page (Figure 2).

### Linking QTLs to the genome and genetic maps

A crucial feature of the solQTL tool is that it links the putative QTL regions to relevant genetic and genomic data in the SGN database. In this subsection, we describe the options for linking the predicted QTLs with other datasets both at SGN and at external databases using bioinformatic tools such as the SGN Comparative Map [39] Viewer and GBrowse [42] tools.

**Figure 3:**
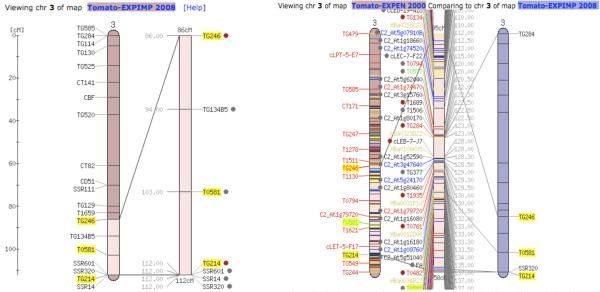
**Comparative genetic analysis of a predicted QTL with other genetic maps**. Panel (A) shows an example linkage map with a zoomed out QTL segment, generated after clicking the linkage group link on the detail page of the QTL of interest (see Figure 2). (B) shows a comparison of the QTL shown on (A) to the Tomato-EXPEN 2000 (F2.2000) consensus genetic map. The zoomed out region, generated after manually feeding the Comparative Map Viewer with the QTL marker positions on the F2.2000 map instead of their genetic positions in the Tomato-EXIMP 2008 map, displays greater number of markers from the reference map within the shared genetic segment between the two maps.

On the QTL detail page (Figure 2) for each QTL, the peak and flanking markers with their corresponding positions on the respective genetic map for the population are shown. The 95% QTL confidence interval is linked to the corresponding linkage group page where one can view all the markers in the linkage group with the QTL segment zoomed out. On the linkage group webpage, users can perform comparative map analysis between the QTL region of interest and physical and genetic maps from several Solanaceae species in the database using the SGN Comparative Map Viewer, as long as the QTL flanking markers are mapped to the other maps. Marker-dense genetic maps available in SGN for comparative analysis include the *S. lycopersicum *LA925 x *S. pennellii *LA716 F2.2000 map [43] which contains more than 2500 Restriction Fragment Length Polymorphism (RFLP), Cleaved Amplified Polymorphic Sequences (CAPS) and Conserved Ortholog Set (COS) markers (Figure 3). The SGN Comparative Map Viewer is described in [39] and additional help and documentation for this tool can be found at http://solgenomics.net/help/cview.pl.

**Figure 4:**
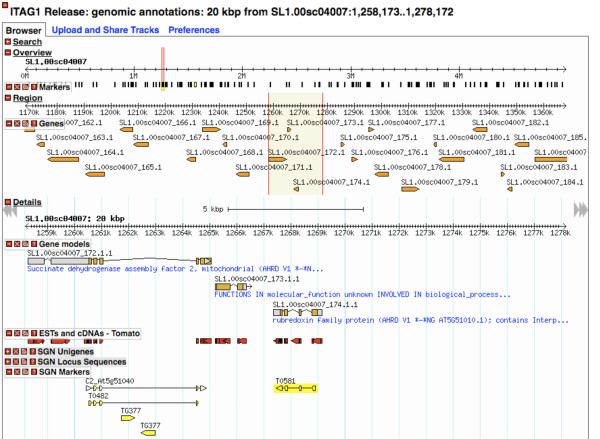
**Linking predicted QTLs to an annotated genome**. An example of a 20 kbp segment on the tomato genome matching a QTL marker (eg. TO581, peak marker for a putative 'Fruit Area' QTL, generated after following a link from the QTL detail page (see Figure 2)). Within the 20 kbp of the marker are annotations including gene and protein models with their GO annotations, ESTs, cDNAs and markers.

From the QTL detail page, the markers are linked to genomic regions visible in SGN's installation of GBrowse, provided that there is a match, where one can view the corresponding genomic sequences and annotations such as gene, mRNA, protein, and CDS models, ESTs, and unigene alignments and experimentally characterized genes from tomato or other organisms (Figure 4). Documentation using GBowse can be found in http://solgenomics.net/gbrowse/static/general_help.html and [42,44].

The peak and flanking markers are also cross-referenced to their respective SGN detail pages where one can find, among other data, the primer sequences, map positions on any other genetic maps and matching BAC clones. For COS markers, one can also find matching Arabidopsis BAC clones and protein hits from NCBI http://www.ncbi.nlm.nih.gov/.

### solQTL tool and data accessibility

solQTL is accessible at http://solgenomics.net/qtl/ or from the 'search' and 'tools' pull down menus on the SGN toolbar, located at the top of every SGN page. From the solQTL database frontpage, users interested in submitting new QTL data and in performing QTL mapping and linking to relevant datasets in SGN can follow the self-guiding 'Submit new QTL data' link. From the same solQTL front page, users intending to query the database for QTLs for traits of interest can also: (1) browse the alphabetically indexed list of traits; (2) use the search box and query the database using relevant keywords; or (3) browse through the list of QTL populations for traits of interest. In the cases of option 1 and 2, the user is directed to the trait's detail page where, among other data, QTL populations assayed for the trait are listed. Following the link to the population of interest initiates solQTL's on-the-fly QTL mapping and generates the QTL detail page for the trait of interest in that QTL population. In the case of option 3, users can select a trait of their interest evaluated in any of the populations and run the QTL mapping analysis.

The population genetic map can be accessed either by following a genetic map link at the population or QTL detail pages or by browsing for it from the 'maps' pull down menu on any SGN page.

## Discussion

### Beyond QTL: comparative analysis and identification of candidate genes

Linking QTLs to corresponding regions in genomes and their annotations is pursued by several biological databases [24,27]. Most databases store pre-computed QTL coordinates, extracted from published literature, and are mostly populated manually by in-house curators. In contrast, SGN has implemented a new approach that dynamically links phenotypes to genomes by implementing the R/QTL mapping software [15] and thus, allowing researchers to upload their raw phenotype and genotype QTL data to the SGN database and set the statistical parameters for the analysis using a user-friendly web interface. To our knowledge, none of these features are available at other databases.

R/QTL is a robust and widely used (with about 450 citations as of this publication, GoogleScholar) open source QTL mapping software. We have implemented its analytical functions such as single marker analysis and interval mapping methods (maximum likelihood [32,33], Haley-Knott regression [34], and Multiple Imputation [35]) for diploid F2 intercross and backcross populations. solQTL is publicly and freely available, and SGN maintains and upgrades the software so that the latest versions are available to run on SGN web servers. In this sense, from the users perspective, the data are stored and analyzed in the 'cloud', as such internet-based services are sometimes referred to, although in this case the 'cloud' is currently limited to the SGN cluster infrastructure. The user-friendly web interface for uploading raw data and setting the statistical parameters combined with the on the-fly QTL analysis by users, in private or public mode, further reinforces the "community annotation" philosophy SGN has pioneered for its locus and phenotype databases [45].

SGN is a clade-oriented database with a computational infrastructure and capacity to store and make accessible data, in a comparative manner, for the Solanaceae family and related Asterids. It is a primary repository for genetic, phenotype, genome and expression data for the Solanaceae family [14]. These datasets are deeply and extensively cross-referenced. As a result, provided that the QTL flanking and/or peak markers exist with their sequences in the SGN marker database, the solQTL tool links dynamically predicted QTLs to corresponding relevant datasets and builds upon the constantly growing networking of emerging datasets in SGN and external databases.

Predicted QTLs are seamlessly cross-linked to the SGN Comparative Map Viewer [39] and SGN's instance of GBrowse [42]. The comparative map viewer enables comparative genetic analysis between QTLs of interest and corresponding regions in genetic and physical maps of the same or different Solanaceae species and Arabidopsis. Corresponding regions in other maps, for example the tomato F2.2000 map [43], with more dense and informative markers such as COS, help identify more tightly linked genetic markers and possibly matching genomic clones or regions. QTLs are currently linked to the GBrowse on a marker-by-marker basis. The GBrowse is also searchable using other genetic features such as unigenes which might enable investigators to infer some missing links from genetic markers to the genome. We anticipate, in the near future, further progress in the assembly of the genomes SGN hosts and search features under development will allow searching for genomic regions matching an entire QTL region. At this stage once a genome region matches a flanking or peak QTL marker, one can zoom in/out (bi-directionally) or scroll the genomic segment one-directionally to explore genomic annotations such as gene, mRNA, CDS and protein models, unigenes, ESTs and experimentally characterized loci. Currently, SGN's GBrowse database houses the *Solanum lycopersicum *cv Heinz 1706 genome sequence and annotations by ITAG [21]. More genomes are in the pipeline to be sequenced and annotated and/or added to SGN's GBrowse and cross-referenced to solQTL.

The solQTL tool allows users to use non-controlled vocabularies trait names. However, users are highly encouraged to use controlled vocabularies for their trait names to facilitate cross-study comparisons for QTLs of the same traits and to fully exploit expression and phenotype data annotated with controlled vocabulary such as the Gene Ontology (GO) [46] and Plant Ontology (PO) [47] terms. SGN in collaboration with the Solanaceae breeders continues to develop and maintain a controlled vocabulary for Solanaceae traits, called Solanaceae Phenotype Ontology, accessible at http://solgenomics.net/tools/onto/.

Looking toward a longer time horizon, the SOL-100 project has an ambitious plan to sequence and make publicly available genomes of 100 or more Solanaceae species [22]. A single nucleotide polymorphism (SNP) database and viewer is also under development at SGN (unpublished). As these projects bear fruit, solQTL will be a window linking phenotypes to this rich genomic data and analytical tools. We envision this integration and analysis of a wide variety of data in SGN and cross-referenced external databases will have an important role to play in identification of candidate genes for further functional analysis and gene cloning, identification of more closely linked QTL markers for marker-assisted breeding and understanding the molecular and physiological underlying phenotypic variation for traits of interest.

## Conclusion and future plans

As genome sequencing becomes less costly, the number of organisms with sequenced and annotated genomes will increase. Linking the emerging wealth of genome data to phenotypes in order to help elucidate the molecular basis for phenotypic variation of quantitative traits will be a major challenge. We believe tools such as solQTL will help facilitate the dynamic linking of phenotypes to genomes for the Solanceae species.

At this stage, solQTL implements a limited number of QTL mapping capabilities of the R/QTL software. In the near future, we plan to implement the full capacity of the R/QTL tool such as genetic map estimation, two-dimensional two-QTL scan, QTL mapping for out-crossing and recombinant inbred lines, and account for covariates such as environment. We plan to extend its analysis capabilities to support multiple QTL mapping, QTL effects calculation, and interactive statistical analysis where users can predict QTLs using several combinations of statistical parameters.

We also plan to implement tools to allow meta-QTL analysis across multiple QTL populations. To enable communication between QTL databases and access to the raw and analyzed QTL data at SGN programmatically, we will generate QTL output in XML format as specified by the emerging standards of Minimum Information for QTLs and Association Studies (MIQAS)[48].

## Availability and requirements

All SGN source code and database schemas are open source and available for download at http://github.com/solgenomics. Raw phenotype and genotype data can also be freely downloaded from the SGN web page of the QTL population of interest, provided that the owner has released the data for the public use. solQTL works with all web browsers and good internet connection.

## Authors' contributions

IYT designed the workflow, implemented the R/QTL code and wrote most of the code and part of the solQTL database schema or implemented modules contributed by NM, RMB, LAM and CPAN authors. NM, RMB, and LAM contributed in code writing, database schema development and discussion. LAM oversaw the development of the project. EVDK contributed the QTL data. The manuscript was drafted by IYT and all authors reviewed and contributed up to the final version.

## Supplementary Material

Additional file 1 **Web interface for uploading raw QTL data and statistical parameters**. Web interface for step-wise uploading of QTL population details, list of traits, phenotype data, genotype data and statistical parameters. The user is prompted for the next step after successfully uploading data in the preceding step http://solgenomics.net/phenome/qtl_form.pl. Click here for file

Additional file 2 **QTL population detail page**. A webpage displaying a QTL population's description, list of traits evaluated for QTLs, and their corresponding phenotype data descriptive statistics and links to the QTL analysis page. Clicking on the graph icon initiates the on-the-fly QTL mapping of the trait (eg. for 'Fruit Area', see Figure 1). Also on the same page are functions for downloading the phenotype and genotype data of the population and links to the genetic map and publication of the population (source: http://solgenomics.net/population.pl?population_id=12). Note: the population genetic map shown is a consensus map that included linkage map data from this population. Click here for file
