# Supplementary figures and images for: solQTL: a tool for QTL analysis, visualization and linking to genomes at SGN database
Source: BMC Bioinformatics. 2010 Oct 21;11:525. doi: 10.1186/1471-2105-11-525 (PMC2984588; doi:10.1186/1471-2105-11-525)

## Slide 1
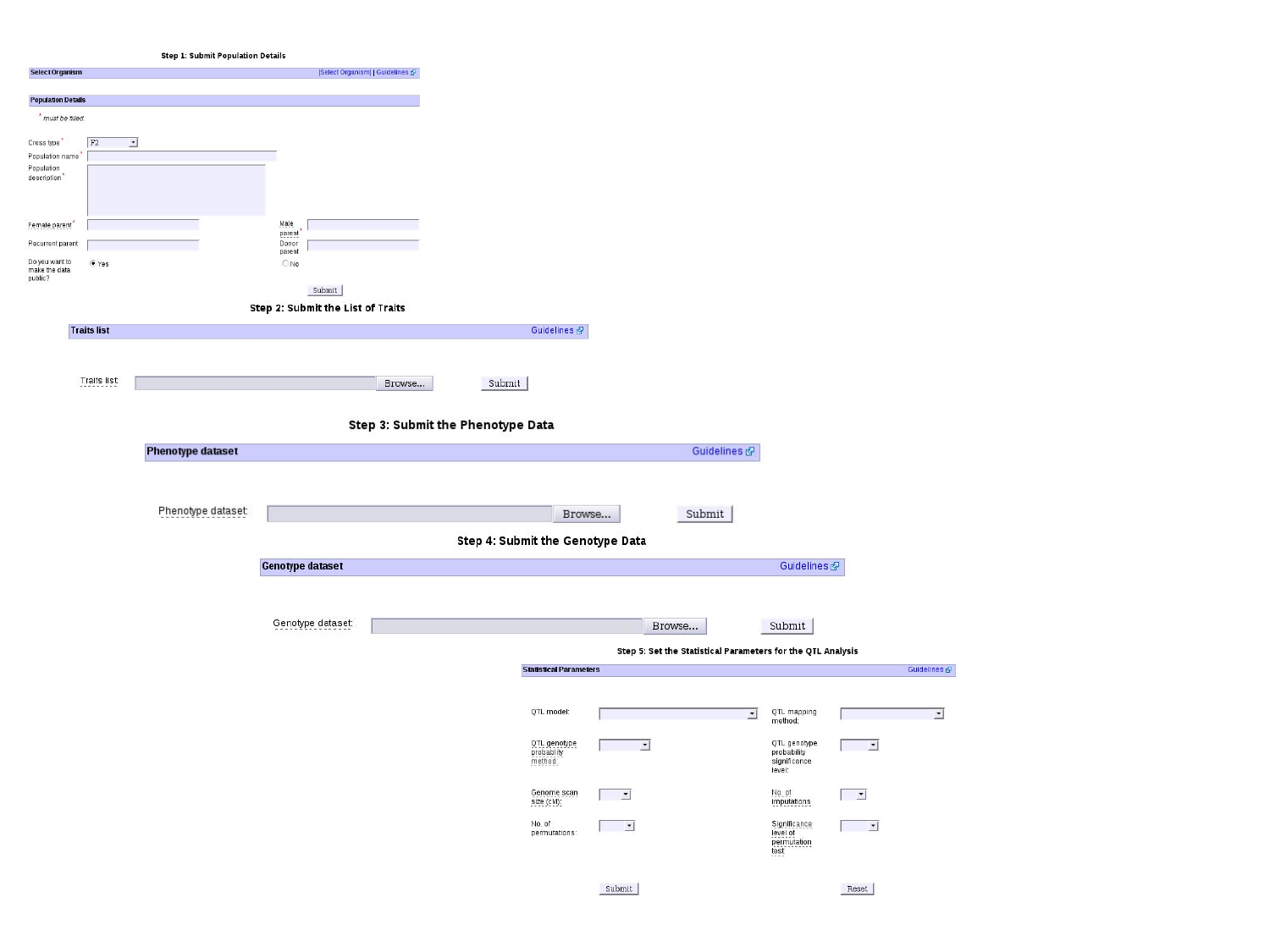

Supplement: Additional file 1 — Web interface for uploading raw QTL data and statistical parameters. Web interface for step-wise uploading of QTL population details, list of traits, phenotype data, genotype data and statistical parameters. The user is prompted for the next step after successfully uploading data in the preceding step http://solgenomics.net/phenome/qtl_form.pl. [file 1471-2105-11-525-S1.PPT]

## Slide 1
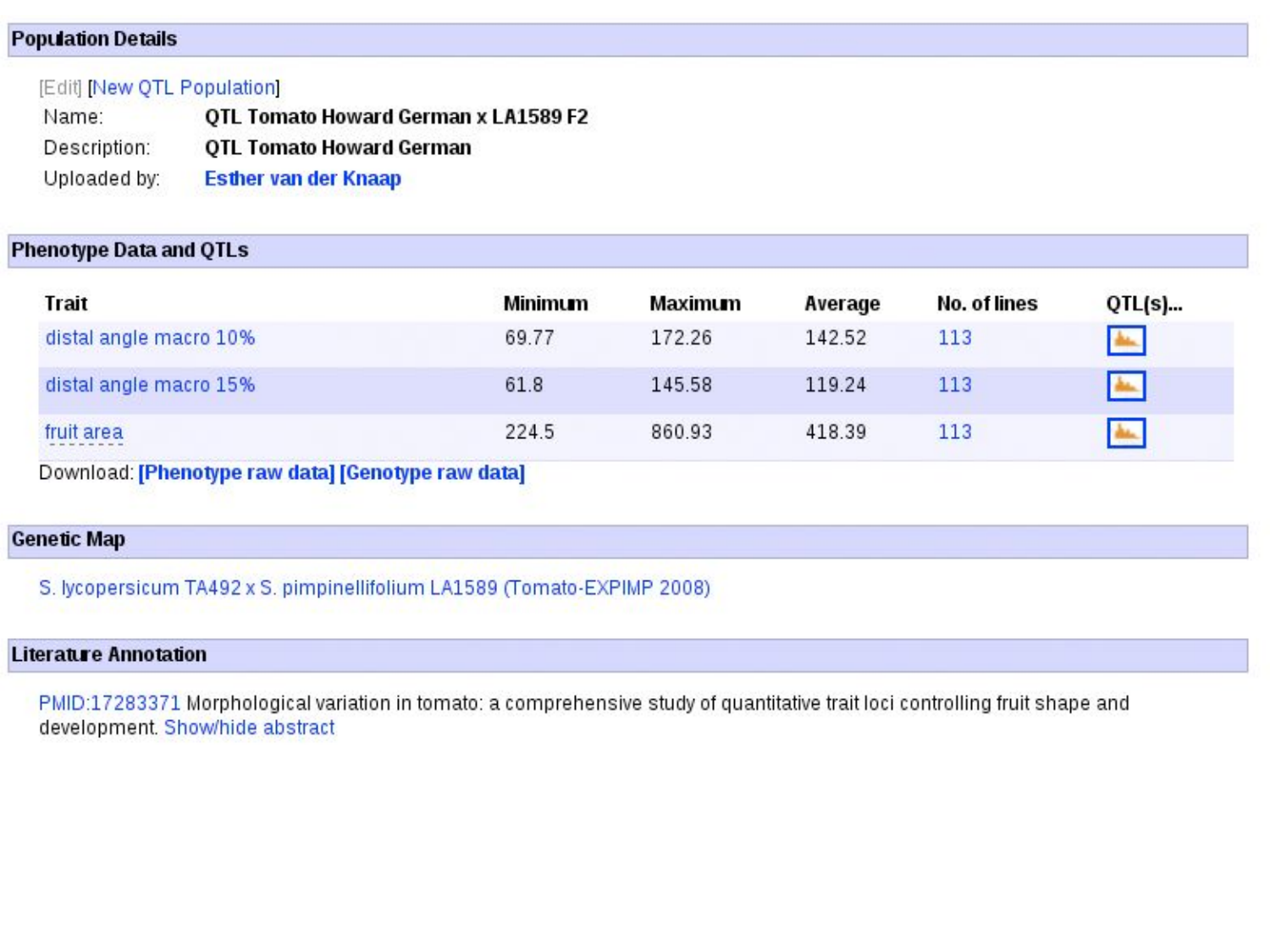

Supplement: Additional file 2 — QTL population detail page. A webpage displaying a QTL population's description, list of traits evaluated for QTLs, and their corresponding phenotype data descriptive statistics and links to the QTL analysis page. Clicking on the graph icon initiates the on-the-fly QTL mapping of the trait (eg. for 'Fruit Area', see Figure 1). Also on the same page are functions for downloading the phenotype and genotype data of the population and links to the genetic map and publication of the population (source: http://solgenomics.net/population.pl?population_id=12). Note: the population genetic map shown is a consensus map that included linkage map data from this population. [file 1471-2105-11-525-S2.PPT]
